# Heavy Metals Pollution and Pb Isotopic Signatures in Surface Sediments Collected from Bohai Bay, North China

**DOI:** 10.1155/2014/158796

**Published:** 2014-04-10

**Authors:** Bo Gao, Jin Lu, Hong Hao, Shuhua Yin, Xiao Yu, Qiwen Wang, Ke Sun

**Affiliations:** ^1^State Key Laboratory of Simulation and Regulation of Water Cycle in River Basin, China Institute of Water Resources and Hydropower Research, Beijing 100038, China; ^2^Department of Water Environment, China Institute of Water Resources and Hydropower Research, Beijing 100038, China; ^3^State Key Laboratory of Water Environment Simulation, School of Environment, Beijing Normal University, Beijing 100875, China

## Abstract

To investigate the characteristics and potential sources of heavy metals pollution, surface sediments collected from Bohai Bay, North China, were analyzed for the selected metals (Cd, Cr, Cu, Ni, Pb, and Zn). The Geoaccumulation Index was used to assess the level of heavy metal pollution. Pb isotopic compositions in sediments were also measured to effectively identify the potential Pb sources. The results showed that the average concentrations of Cd, Cr, Cu, Ni, Pb, and Zn were 0.15, 79.73, 28.70, 36.56, 25.63, and 72.83 mg/kg, respectively. The mean concentrations of the studied metals were slightly higher than the background values. However, the heavy metals concentrations in surface sediments in Bohai Bay were below the other important bays or estuaries in China. The assessment by Geoaccumulation Index indicated that Cr, Zn, and Cd were classified as “the unpolluted” level, while Ni, Cu, and Pb were ranked as “unpolluted to moderately polluted” level. The order of pollution level of heavy metals was: Pb > Ni > Cu > Cr > Zn > Cd. The Pb isotopic ratios in surface sediments varied from 1.159 to 1.185 for ^206^Pb/^207^Pb and from 2.456 to 2.482 for ^208^Pb/^207^Pb. Compared with Pb isotopic radios in other sources, Pb contaminations in the surface sediments of Bohai Bay may be controlled by the mix process of coal combustion, aerosol particles deposition, and natural sources.

## 1. Introduction

Bohai Bay is a semienclosed bay located in the western region of the Bohai Sea and is one of the four major compositions in Bohai Sea, North China. It covers an area of 1.60 × 104 km^2^ with a population of about 70 million. The average depth is 12.5 m with the maximum of 32 m. Bohai Bay is important harbor and marine lines of economic development in North China. With the rapid development of urban economy and industry around sea, Bohai Bay receives both industrial and domestic wastewater discharges from Beijing, Tianjin, and Hebei province. All the wastewater through rivers and channels drains into the near-shore waters of Bohai Bay directly. This process produced a certain degree of heavy metal pollution for ocean environment [1]. In fact, sediments can receive and absorb metal pollutants from natural weathering, erosion, and anthropogenic activities. Heavy metal concentrations in ocean sediments are important indicators which can reflect the heavy metal pollution of ocean environment [2]. Moreover, heavy metals may be recycled via chemical and biological processes within these sedimentary compartment and back to the water column [3]. The accumulation of metal contaminants in sediments can pose serious environmental problems to the ocean environment. Therefore, identifying the sources of heavy metals is of key importance for making decisions concerning site remediation. Up till now, most researchers have identified the metal sources in sediments by using variations of metal concentrations and enrichment factors relative to natural inputs. However, recent studies have proved that the Pb stable isotopic signatures were a useful tool to effectively identify various sources of Pb pollution in water environment [4–6].

In the recent years, the researches of heavy metals in Bohai Bay had mainly focused on the estuarine and the intertidal zone [7–9]. The scale of whole Bay consisting of the estuarine, the intertidal zone, and centre of the Bay was not fully researched for the characteristics of heavy metals pollution. The objectives of this present study were (1) to determine concentrations and distribution characteristics of heavy metals in surface sediments collected from the Bohai Bay, (2) to evaluate the degree of heavy metal contamination, and (3) to identify the metal pollution sources using Pb isotopic signature. We hope our research on heavy metals contamination and Pb isotopic signatures will be useful to assess the environmental impact of urbanization and economic development on ocean water environment, while providing scientific information for environmental management and restoration in this region.

## 2. Materials and Methods

### 2.1. Sampling Sites

**Figure 1:**
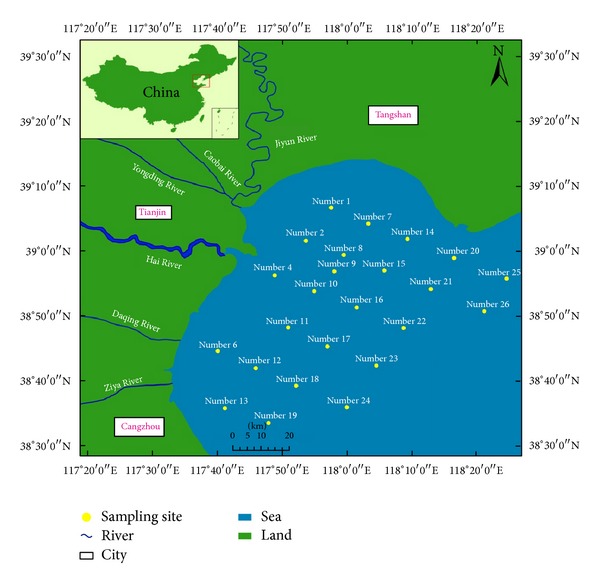
Sampling sites in Bohai Bay.

Eighteen surface sediments (0–10 cm) were collected from Bohai Bay, North China. A map of the sampling sites is shown in Figure 1. After sampling, the sediment samples were taken back to indoor laboratory and dried at a temperature below −40°C, crushed, and sieved to less than 200 *μ*m before the chemical measurements were taken as described below.

### 2.2. Analytical Methods

All chemical treatments were in the ultraclean laboratory, and all reagents were of high purity grade. A strong acid digestion method (HNO_3_ + H_2_O_2_ + HF) was used to dissolve heavy metals in solution [10]. The digested solutions were measured using inductively coupled plasma-mass spectrometry (ICP-MS, Perkin Elmer Elan DRC-e) for the concentrations of Cd, Cr, Cu, Ni, Pb, and Zn. The accuracy of the analytical procedures employed for the analysis of the metals in sediments was checked using the certified reference material of China stream sediment (GSD-12, GBW07312), obtaining good agreement with certified values.

### 2.3. Pb Isotopic Measurement

Pb isotopic analyses was separated using microexchange columns of anion resin of Dowex-I (200–400 mesh) and HBr and HCl as eluants [11]. Measurements of Pb isotopic compositions were carried out using an ICP-MS (Perkin Elmer Elan DRC-e). The average measured values of the standard NIST SRM-981 are ^206^Pb/^207^Pb = 1.0937 ± 0.0012 and ^208^Pb/^207^Pb = 2.3695 ± 0.032 (*n* = 20), respectively, which were in close agreement with the certified standard values (1.0933 and 2.3704, resp.). Analytical uncertainties in 2s (2s, 2 standard deviation, *n* = 20) for Pb isotopic ratios (^206^Pb/^207^Pb) were generally <0.5%.

## 3. Results and Discussion

### 3.1. Heavy Metal Concentrations in Surface Sediments

**Figure 2:**
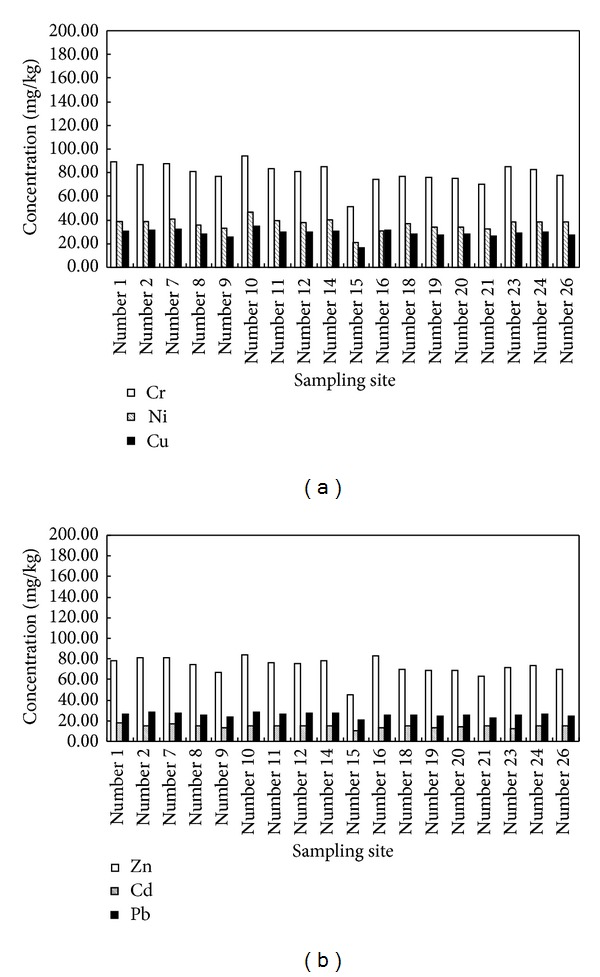
Spatial distribution of heavy metals in surface sediments of Bohai Bay.

The heavy metal concentrations and statistics results of all investigated sediments in Bohai Bay are summarized in Table 1. For the comparison purpose, the background values of heavy metals and heavy metals concentrations in sediments from the other Bays and Estuaries were also shown. As can be seen, the concentration ranges of heavy metals of Cr, Ni, Cu, Zn, Cd, and Pb in Bohai Bay sediments were 51.32~94.33, 21.88~47.04, 16.86~34.99, 45.31~84.19, 0.11~0.18, and 20.69~28.33 mg/kg, respectively; and the mean concentrations of these metals were 79.73, 36.56, 28.70, 72.83, 0.15, and 25.63 mg/kg, respectively. The order of mean concentrations in surface sediments was Cr > Zn > Ni > Cu > Pb > Cd. In the sediments from the Bohai Bay, the mean concentrations of the studied metals were slightly higher than the background values [12]. In addition, low variable coefficient (<15%) of these metals showed that the heavy metals were evenly distributed in the Bohai Bay. In fact, the heavy metals concentrations in surface sediments of Bohai Bay were above those in the intertidal Bohai Bay, Southern Bohai Bay, and Western Bohai Bay, indicating that possible anthropogenic input was in the central part of Bohai Bay. However, heavy metals concentrations in surface sediments in Bohai Bay were below those in the other estuaries in China, such as Yangtze Estuary and Pearl River Estuary. In addition, the spatial distribution of heavy metals in sediments collected from Bohai Bay is shown in Figure 2 (Cd concentration is 100 times more than the actual concentration). From Figure 2, it can be seen that heavy metal concentrations in sediments close to center of Bay (number 10) were the highest among all sediments in the whole Bay (Figure 1).

Marine sediment quality (GB 18668-2002), established by China State Bureau of Quality and Technical Supervision (CSBTS, 2002), contains three standard criteria for marine sediments. The marine sediment quality I is applied to protect the habitats for marine life including natural, rare, and endangered species as well as the areas for human recreation and sports, while the marine sediment quality II is applied to regulating general industrial use and coastal tourism. Based on marine sediment quality (GB 18668-2002), the overall mean concentrations of all selected heavy metals in Bohai Bay are below those value of the marine sediment quality I, indicating that the overall sediments quality in the Bohai Bay has not been obviously impacted by these six heavy metals. However, the mean concentration of Cr was close to the marine sediment quality I and Cr concentrations in some sampling sites have higher values than the value of marine sediment quality I, especially for the number 10 (Figure 2).

Sediment quality guidelines (SQGs) have been developed to deal with environmental concerns, and a stricter criterion in Hong Kong was also chosen to assess the contamination level of individual metals in sediments of the Bohai Bay (Table 1) [13]. The concentrations of Cu, Pb Zn, and Cd in sediments from 94.4%, 100%, 44.4%, and 100% of stations, respectively, were lower than “target” values and showed no signs of contamination. The contents of Cr at 100% of stations and Ni at 66.7% of stations were higher than the “trigger” values which are regarded as the upper limit of the desired quality for fairly clean sediments, indicating Bohai Bay sediments were moderately contaminated for Cr and Ni. In fact, there were 55.6% and 16.7% sediments heavily polluted by Cr and Ni (Figure 2 and Table 1).

### 3.2. Pollution Assessment

The Geoaccumulation Index (*I*_geo_) introduced by Müller (1979) was used to assess metal pollution in sediments of Bohai Bay [14]. Geoaccumulation Index is expressed as follows:

(1) $$\begin{array}{c} I_{\text{geo}}=\log_{2}\left(\frac{C_{n}}{1.5B_{n}}\right), \end{array}$$

where *C*_*n*_ is the measured concentration of heavy metal (*n*) in the sediment, *B*_*n*_ is the geochemical background value of heavy metal (*n*), and 1.5 is the background matrix correction factor due to lithogenic effects. In the present study, *B*_*n*_ was selected from the literature [15]. Geoaccumulation Index includes seven grades from Class 0 (*I*_geo_ ≤ 0) to Class 6 (*I*_geo_ ≥ 5). The *I*_geo_ is associated with a qualitative scale of pollution intensity; samples may be classified as unpolluted (*I*_geo_ ≤ 0), unpolluted to moderately polluted (0 ≤ *I*_geo_ ≤ 1), moderately polluted (1 ≤ *I*_geo_ ≤ 2), moderate to strongly polluted (2 ≤ *I*_geo_ ≤ 3), strongly polluted (3 ≤ *I*_geo_ ≤ 4), strongly to extremely polluted (4 ≤ *I*_geo_ ≤ 5), and extremely polluted (*I*_geo_ ≥ 5).

Based on the *I*_geo_ data and the Geoaccumulation Index, the results of the *I*_geo_ values and pollution level of heavy metals of surface sediment in Bohai Bay are shown in Table 2. In general, the average *I*_geo_ values are −0.91 for Cr, 0.27 for Ni, 0.11 for Cu, −0.20 for Zn, −0.53 for Cd, and 0.59 for Pb. The order of average *I*_geo_ values was Pb > Ni > Cu > Cr > Zn > Cd. Among the average *I*_geo_ of Cr, Zn and Cd were less than zero (*I*_geo_ ≤ 0), which were classified as “unpolluted” level. However, the *I*_geo_ value of Cr and Zn in sampling site number 10 was more than zero, which were ranked as “unpolluted to moderately polluted” level. In addition, the average *I*_geo_ values of Ni, Cu, and Pb were ranked as “unpolluted to moderately polluted” level (0 ≤ *I*_geo_ ≤ 1). However, the *I*_geo_ value of Ni and Cu in sampling site number 15 was less than zero. In general, the worst pollution of heavy metals occurred in sampling site number 10 and the lightest pollution of heavy metals was in sampling site number 15.

### 3.3. Pb Isotopic Compositions in Surface Sediments

The results of Pb isotope ratios of sediment samples in Bohai Bay and other environmental samples (natural sources, ores in this region, vehicle exhaust, aerosol samples, and unburned coal) are shown in Table 3. Lead isotopic ratios ranged from 1.159 to 1.185 for ^206^Pb/^207^Pb and 2.456 to 2.482 for ^208^Pb/^207^Pb. From Table 3, it can be seen that sediments from all sampling sites have relative lower values of ^206^Pb/^207^Pb ratios close to those values in pollution sources, suggesting the influence of anthropogenic inputs in Bohai Bay. In order to assess Pb contamination and identify potential Pb sources of sediments, the correlation between 1/Pb concentrations and ^206^Pb/^207^Pb ratios were analyzed. The analytic results showed that the relationship was not obviously correlated (*R* ^2^ = 0.2772), indicating that Pb sources of sediments were relatively complicated and cannot be simply attributed to binary mixing process of two sources [6].

**Figure 3:**
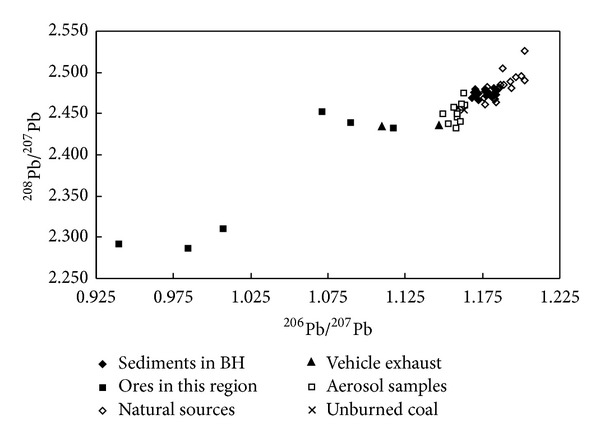
Comparison of the Pb isotopic ratios in sediments of Bohai Bay and other sources.

Source apportionment can also be accomplished by analyzing the Pb isotopic radios (^206^Pb/^207^Pb and ^208^Pb/^207^Pb) in the environmental samples and major pollution sources with a lines mixing model [6]. The comparison between ^206^Pb/^207^Pb and ^208^Pb/^207^Pb ratios in sediments and other environmental samples showed that the ^206^Pb/^207^Pb ratios in surface sediments collected from Bohai Bay were obviously higher than those from the emission of vehicle exhaust and Pb ores mining, indicating that these two anthropogenic inputs were not important factors for Pb pollution in sediments (Figure 3). Wang et al. also have confirmed that lead concentrations of atmospheric aerosols in Tianjin were decreased significantly after the leaded gasoline ban [16]. In fact, the ^206^Pb/^207^Pb and ^208^Pb/^207^Pb ratios in sediments were significantly similar to those in natural sources, unburned coal, and aerosol samples in the city of Tianjin. In the northern part of China, coal became one of most important energy resources in urban economical and industrial development. The leaded particular matter from coal combustion and coupled with the urban dusts (contain a mass of cement material) can be transported to the Bohai Bay by atmospheric deposition and surface river runoff. Previous study has shown that a large amount of Pb was supplied by the precipitation of aerosols in coastal environments and coal burning from power generation plants and other industrial activities may be the major source of Pb in its sediments [17]. Therefore, Pb contamination in the sediments of Bohai Bay may be controlled by the mix process of coal combustion, aerosol particles deposition, and natural sources.

## 4. Conclusion

Our investigation of heavy metals (Cr, Ni, Cu, Zn, Cd, and Pb) in surface sediments collected from Bohai Bay showed that the mean concentrations of the studied metals were slightly higher than the background values. However, the heavy metals concentrations in surface sediments in Bohai Bay were below the other important bays or estuaries in China. The assessment by Geoaccumulation Index indicated that Cr, Zn, and Cd were at the “unpolluted” level, while Ni, Cu, and Pb were ranked as “unpolluted to moderately polluted” level. The pollution level of the heavy mental was Pb > Ni > Cu > Cr > Zn > Cd. The Pb isotopic ratios in surface sediments varied from 1.159 to 1.185 for ^206^Pb/^207^Pb and from 2.456 to 2.482 for ^208^Pb/^207^Pb. Pb sources in sediments from Bohai Bay were more complicated (more than two sources), and there may exist various pollution sources. Compared with the Pb isotopic radios in other sources, coal combustion, aerosol particles deposition, and natural sources may be the major sources for Pb pollution in surface sediments of Bohai Bay.

## Figures and Tables

**Table 1:** Heavy metal concentrations in surface sediments of Bohai Bay and other Bays in China (mg/kg).

Station	Cr	Ni	Cu	Zn	Cd	Pb	References
Minimum	51.32	21.88	16.84	45.31	0.11	20.69	This study
Maximum	94.33	47.04	34.99	84.19	0.18	28.33	
Mean	79.73	36.56	28.70	72.83	0.15	25.63	
Variable coefficient (%)	11.39	13.84	12.71	12.04	10.29	7.25	
Intertidal Bohai Bay, China	68.6	28.0	24.0	73.0	0.12	25.6	[5]
Southern Bohai Bay, China	33.5	30.5	22.7	71.7	0.14	21.7	[9]
Western Bohai Bay, China	53.1	31.4	27.9	83.6	0.13	20.5	[8]
Upper continental crust	35	20	25	71	0.098	16.6	[12]
Yangtze Estuary, China	78.9	31.8	30.7	94.3	0.26	27.3	[18]
Pearl River Estuary, China	89.0	41.7	46.2	150	n.d.	59.3	[19]
Marine sediment quality I	80	n.d.	35	150	0.5	60	[20]
Marine sediment quality II	150	n.d.	100	350	1.5	130	[20]
Target*	25	20	20	75	0.4	35	[13]
Trigger*	50	35	55	150	1.0	65	[13]
Action*	80	40	65	200	1.5	75	[13]

*Target indicates the desired quality for fairly clean sediment that is close to background levels.

Trigger indicates that the sediment is moderately contaminated.

Action indicates heavily polluted sediments.

**Table 2:** *I*_geo_ values of heavy metals in surface sediments of Bohai Bay.

Sampling sites	Cr	Ni	Cu	Zn	Cd	Pb
1#	−0.01/0	0.36/1	0.23/1	−0.08/0	−0.24/0	0.66/1
2#	−0.05/0	0.39/1	0.24/1	−0.03/0	−0.47/0	0.73/1
7#	−0.03/0	0.46/1	0.31/1	−0.04/0	−0.34/0	0.70/1
8#	−0.15/0	0.27/1	0.11/1	−0.16/0	−0.49/0	0.57/1
9#	−0.23/0	0.13/1	−0.03/0	−0.30/0	−0.62/0	0.50/1
10#	0.07/1	0.65/1	0.41/1	0.02/1	−0.50/0	0.74/1
11#	−0.11/0	0.39/1	0.17/1	−0.12/0	−0.44/0	0.62/1
12#	−0.15/0	0.35/1	0.16/1	−0.14/0	−0.44/0	0.67/1
14#	−0.09/0	0.42/1	0.20/1	−0.08/0	−0.46/0	0.68/1
15#	−0.81/0	−0.46/0	−0.64/0	−0.87/0	−0.98/0	0.29/1
16#	−0.28/0	0.04/1	0.27/1	0.00/0	−0.64/0	0.61/1
18#	−0.22/0	0.26/1	0.09/1	−0.25/0	−0.46/0	0.58/1
19#	−0.25/0	0.22/1	0.04/1	−0.27/0	−0.67/0	0.54/1
20#	−0.25/0	0.21/1	0.08/1	−0.27/0	−0.54/0	0.60/1
21#	−0.35/0	0.11/1	−0.01/0	−0.39/0	−0.49/0	0.41/1
23#	−0.08/0	0.37/1	0.15/1	−0.20/0	−0.72/0	0.61/1
24#	−0.12/0	0.35/1	0.16/1	−0.17/0	−0.49/0	0.62/1
26#	−0.22/0	0.31/1	0.07/1	−0.25/0	−0.46/0	0.55/1

Average	−0.19/0	0.27/1	0.11/1	−0.20/0	−0.53/0	0.59/1

**Table 3:** Comparison of Pb isotope ratios of surface sediments in Bohai and other sources in environment.

Sample	^ 206^Pb/^207^Pb	^ 208^Pb/^207^Pb	Reference
Sediments in Bohai Bay	1.159–1.185 (average 1.176)	2.456–2.482 (average 2.473)	In this study
Natural sources	1.178–1.202	2.462–2.526	In this study
Ores in this region	1.143–1.205	2.446–2.494	[21]
Vehicle exhaust (leaded)	1.099	2.4349	[22]
Vehicle exhaust (unleaded)	1.1468	2.4358	[22]
Unburned coal	1.1628	2.4548	[22]
Air deposition	1.150–1.194	2.428–2.485	[16]
